# Learning robust and generalizable bimanual skills: a spatiotemporal causal hierarchical diffusion framework with attention anti-interference

**DOI:** 10.3389/fnbot.2026.1849143

**Published:** 2026-06-22

**Authors:** Xukun Liu, Fengjuan Xie, Zhenyu Liu, Guangning Li, Shenggang Wei, Kai Xu, Aifeng Liu

**Affiliations:** Northwest Institute of Mechanical and Electrical Engineering, Xianyang, Shaanxi, China

**Keywords:** attention anti-interference, bimanual visuomotor imitation, causal representation learning, hierarchical diffusion policy, robust manipulation

## Abstract

**Introduction:**

Bimanual visuomotor imitation learning enables robots to acquire coordinated dual-arm manipulation skills from visual demonstrations, yet it faces significant challenges in temporal synchronization, spatial collision avoidance, long-horizon reasoning, and robustness to visual distractions. Existing diffusion-based policies often struggle to simultaneously capture long-horizon temporal dependencies and fine-grained spatial precision, while remaining sensitive to spurious correlations and domain shifts.

**Methods:**

To address these limitations, we propose the Spatiotemporal Causal Hierarchical Diffusion Imitation Learner (SCH-DIL), a framework that integrates spatiotemporal hierarchical diffusion optimization to factorize the denoising process into temporal and spatial branches for multi-scale action modeling. The framework further incorporates causal visual representation learning that minimizes mutual information with confounding environmental factors to produce invariant features, along with noise-robust diffusion modeling that employs learnable observation uncertainty estimation and confidence-aware denoising. Additionally, attention anti-interference regularization is introduced to penalize distractions and enforce temporal attention consistency.

**Results:**

Extensive experiments on the RoboTwin 2.0 benchmark demonstrate that SCH-DIL consistently outperforms existing diffusion-based and imitation learning baselines, achieving higher success rates under both clean and domain-randomized inference settings.

**Discussion:**

These improvements are achieved with minimal computational overhead, suggesting that the proposed hierarchical and causally regularized diffusion framework offers a practical and robust solution for bimanual visuomotor imitation learning in visually challenging and domain-shifted environments.

## 1 Introduction

Bimanual visuomotor imitation learning aims to enable robots to acquire coordinated dual-arm manipulation skills from visual demonstrations. Compared to single-arm settings, bimanual tasks impose additional challenges, including temporal synchronization between arms, spatial collision avoidance, and long-horizon reasoning under partial observability. While recent diffusion-based policies have shown promise in modeling multi-modal action distributions and improving robustness over deterministic behavior cloning, they still face critical limitations when applied to realistic bimanual scenarios.

A primary challenge lies in the inability of standard diffusion policies to simultaneously capture long-horizon temporal dependencies and fine-grained spatial precision. Most existing approaches treat action generation as a monolithic denoising process, which either loses high-level temporal structure or fails to preserve low-level control accuracy. This limitation becomes particularly severe in tasks requiring both global coordination and local refinement over extended time horizons.

Another major obstacle is the sensitivity of visual representations to spurious correlations and domain shifts. Real-world deployments involve varying lighting conditions and cluttered backgrounds. Conventional policies that rely on correlation-based visual features often degrade significantly under such perturbations.

To address these challenges, we propose Spatiotemporal Causal Hierarchical Diffusion Imitation Learner (SCH-DIL), a novel framework for robust and generalizable bimanual visuomotor imitation. Our method introduces four key innovations:

- Spatiotemporal hierarchical diffusion optimization that factorizes the denoising process into a temporal diffusion branch for high-level action rhythms and a spatial diffusion branch for low-level control precision, enabling joint modeling of multi-scale dependencies.
- Causal visual representation learning that explicitly minimizes mutual information between learned features and confounding environment factors, producing invariant representations robust to visual distractions.
- Noise-robust diffusion modeling with learnable observation uncertainty estimation and confidence-aware denoising, enhancing policy stability under noisy observations and action stochasticity.
- Attention anti-interference regularization that penalizes attention allocated to non-task-relevant regions and enforces temporal attention consistency, reducing distraction in cluttered or occluded scenes.

We evaluate SCH-DIL on the RoboTwin 2.0 benchmark, a challenging bimanual manipulation platform with dynamic scenes, dual-arm coordination, and strong domain randomization. Experimental results demonstrate that our method consistently outperforms existing diffusion-based and imitation learning baselines, achieving higher success rates under both clean and randomized inference settings with minimal computational overhead.

## 2 Related work

Diffusion-based visuomotor policy learning has recently emerged as a powerful paradigm for robot control, offering a flexible alternative to conventional behavior cloning and reinforcement learning approaches. Early works such as Diffusion Policy model action generation as a conditional denoising process, enabling the policy to capture complex, multimodal action distributions and improving robustness over deterministic mappings (Chi et al., 2025). Recent large-scale diffusion-based robotic foundation models further demonstrate the scalability of generative policies for manipulation. For example, RDT-1B introduces a diffusion transformer architecture for bimanual manipulation with unified action representations across heterogeneous robotic platforms (Liu et al., 2025a), while π0 explores vision-language-action policy learning for general robot control through large-scale multimodal pretraining (Black et al., 2024). Building upon these developments, subsequent methods incorporate structured representations to enhance generalization, such as 3D-aware policies that leverage point cloud features for spatial reasoning (Ze et al., 2024). Meanwhile, efficiency-oriented variants, including distilled or one-step diffusion models, aim to reduce inference latency while maintaining policy expressiveness (Wang et al., 2024). Despite these advances, most existing diffusion-based policies implicitly assume clean observations and are sensitive to visual perturbations, motivating the need for more robust formulations.

To address long-horizon reasoning and complex task structure, hierarchical modeling has been widely studied in robotics and sequential decision-making. Classical approaches decompose tasks into high-level planning and low-level control, enabling temporal abstraction and improved scalability (Xu et al., 2025). Recent learning-based methods extend this idea by incorporating latent skill representations and multi-scale temporal modeling into policy learning frameworks (Willibald and Lee, 2022; Zhao et al., 2023; Keller et al., 2025). Within diffusion-based methods, hierarchical designs have begun to emerge, where coarse-to-fine generation aligns action synthesis with multi-scale visual features (Nandiraju et al., 2025). However, existing approaches typically focus on either spatial hierarchy or temporal modeling in isolation, lacking a unified spatiotemporal formulation across the diffusion process, which limits their effectiveness in dynamically evolving and long-horizon tasks.

Another line of research focuses on improving visual representation learning for robust policy generalization. Conventional visuomotor policies often rely on correlation-based features, which are susceptible to spurious patterns and background noise (Geirhos et al., 2020; Ma et al., 2024; Sikand et al., 2022). To address this issue, recent works explore causality-inspired representation learning, aiming to disentangle invariant task-relevant factors from nuisance variations (Liu et al., 2024; Liang et al., 2026; Cai et al., 2025). Techniques such as invariant risk minimization, causal feature masking, and interventional data augmentation have shown promise in reducing sensitivity to domain shifts and visual distractions (Octo Model Team, 2024; Xia et al., 2025; Hoque et al., 2024). In robotic manipulation, causal representations have been applied to improve object-centric reasoning and generalization across unseen environments (Dai et al., 2025; Dcruz et al., 2024), but their integration with diffusion-based policies remains limited.

Attention mechanisms have also become a core component in modern visuomotor policies, enabling selective focus on task-relevant information across spatial and temporal dimensions (Gervet et al., 2023; Liu et al., 2025b; Li et al., 2020). Transformer-based architectures have demonstrated strong capability in modeling long-range dependencies for sequential decision-making tasks (Cai et al., 2024; Chen et al., 2023; Zhou and Zhang, 2025). Although some attention regularization strategies originated from action recognition and sequential perception, their principles for suppressing irrelevant features and stabilizing temporal attention have been adopted in robotic visuomotor policy learning. Nevertheless, attention modules remain sensitive to noisy and cluttered observations. Recent studies introduce sparsity constraints, consistency losses, and perturbation-aware training to improve attention robustness (Chen et al., 2025; Xu et al., 2022; Zhang et al., 2025), yet these methods remain underexplored in diffusion-based policy frameworks. Beyond robotics, spatio-temporal graph attention networks with regularization for distribution shifts have shown promise in maintaining attention consistency under environmental variations (Wang and Wang, 2016; Wei et al., 2025). Such principles could inform future efforts to reduce reliance on perfect mask annotations in real-world deployment.

Recent work highlights the importance of realistic evaluation platforms for visuomotor policy learning. Benchmarks such as Meta-World (Yu et al., 2020), RLBench (James et al., 2020), and ManiSkill (Li et al., 2024) enable standardized comparison but are limited to clean visual settings and simplified dynamics. Large-scale frameworks like RoboNet (Nagiredla et al., 2024) and Robocasa (Nasiriany et al., 2024) improve data diversity, yet mainly focus on single-arm and static scenarios. More recent platforms such as RoboTwin 2.0 (Chen et al., 2025) introduce dual-arm coordination, dynamic scenes, and significant visual interference, providing a more realistic testbed for evaluating robustness, long-horizon reasoning, and collaborative manipulation in diffusion-based policies.

## 3 Method

**Figure 1:**
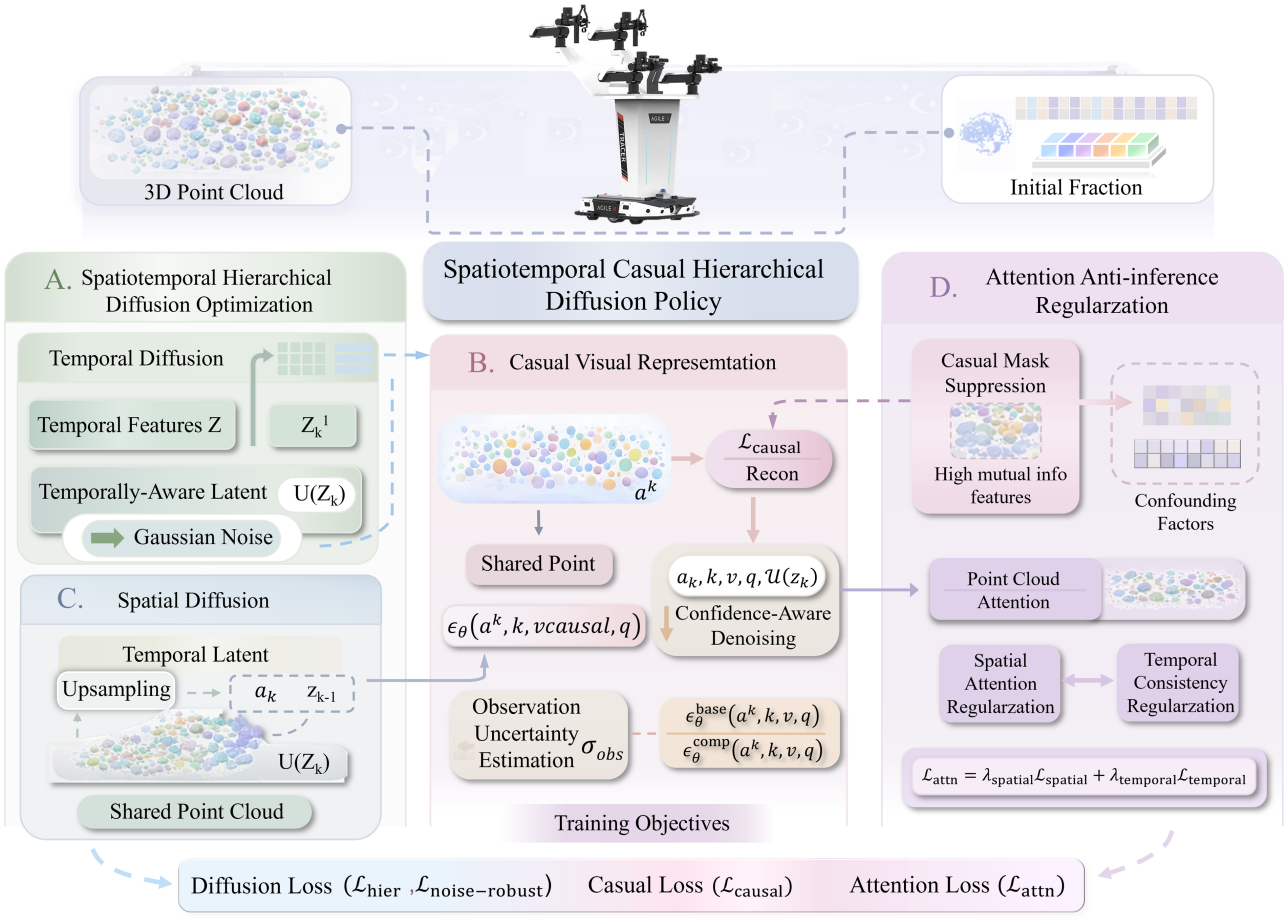
Overall architecture of spatiotemporal causal hierarchical diffusion policy.

We present Spatiotemporal Causal Hierarchical Diffusion Imitation Learner (SCH-DIL), a novel visuomotor imitation learning framework that enhances generalization, robustness, and higher success rates through four key innovations: spatiotemporal hierarchical diffusion optimization, causal visual representation learning, noise-robust diffusion modeling, and attention-based anti-interference regularization. Given only a small set of expert demonstrations, SCH-DIL learns a policy π:O↦A that maps visual observations o∈O to actions a∈A . The overall architecture, illustrated in Figure 1, integrates these components into an end-to-end trainable system.

### 3.1 Spatiotemporal hierarchical diffusion optimization

Standard diffusion policies typically model action sequences as a monolithic denoising process, which struggles to capture multi-scale temporal dependencies and spatial precision. To this end, we introduce a spatiotemporal hierarchical diffusion optimization mechanism that factorizes the denoising process into two complementary levels: a temporal diffusion that models high-level action rhythms, and a spatial diffusion that refines low-level control precision.

Let the action sequence be denoted as $A=[a_{1},a_{2},\ldots ,a_{T}]\in {\mathbb{R}}^{T\times d_{a}},$ where T is the prediction horizon and $\;d_{a}\;$ is the action dimensionality. We decompose the reverse diffusion process into two coupled chains operating at different temporal resolutions.

### 3.2 Temporal diffusion

We introduce a latent variable $\;z\in {\mathbb{R}}^{T^{\prime }\times d_{z}}$ with $\;T^{\prime }<T$ to represent temporal abstractions. The temporal denoising network $\in_{\theta }^{\text{temp}}$ learns to generate z conditioned on the 3D visual feature v and robot state q (Equation 1):


$$z_{k-1}=\alpha_{k}^{\text{temp}}(z_{k}-\gamma_{k}^{\text{temp}}\in_{\theta }^{\text{temp}}(z_{k},k,v,q))+\sigma_{k}^{\text{temp}}N(0,I)$$ (1)

Where k denotes the diffusion timestep, $\alpha_{k}^{\text{temp}},\gamma_{k}^{\text{temp}},\sigma_{k}^{\text{temp}}$ are noise schedule parameters, and N(0,I) is standard Gaussian noise. The temporal latent is upsampled to full resolution via a learnable interpolation operator $U:{\mathbb{R}}^{T^{\prime }\times d_{z}}\to {\mathbb{R}}^{T\times d_{z}}$ (Equation 2).

### 3.3 Spatial diffusion

The fine-grained actions are generated conditioned on both the visual feature and the temporally-aware latent:


$$a_{k-1}=\alpha_{k}^{\text{spat}}(a_{k}-\gamma_{k}^{\text{spat}}\in_{\theta }^{\text{spat}}(a_{k},k,v,q,U(z_{k})))+\sigma_{k}^{\text{spat}}N(0,I)$$ (2)

The joint training objective for the hierarchical diffusion is (Equation 3):


$$\begin{array}{c} L_{\text{hier}}=E_{k,\in^{\text{temp}},\in^{\text{spat}},a^{0}}[\begin{array}{l} {\left\|\in^{\text{temp}}-\in_{\theta }^{\text{temp}}(z_{k},k,v,q)\right\|}_{2}^{2}+\\ {\left\|\;\in^{\text{spat}}-\in_{\theta }^{\text{spat}}(a_{k},k,v,q,U(z_{k}))\right\|}_{2}^{2} \end{array}] \end{array}$$ (3)

where $\in^{\text{temp}}$ and $\in^{\text{spat}}$ are independent Gaussian noise variables sampled for the temporal and spatial diffusion branches, respectively.

This hierarchical factorization enables the model to capture long-horizon temporal coherence while preserving spatial precision, significantly improving learning efficiency with limited demonstrations.

### 3.4 Causal visual representation

The perception module is designed to extract causal visual features that are invariant to spurious correlations arising from confounding environmental factors (e.g., lighting, background, camera viewpoint). Unlike standard 3D representations that indiscriminately encode all scene information, we propose a causal feature learning framework that separates task-relevant geometric cues from such nuisances.

We adopt point clouds as the primary 3D representation for their geometric richness. Given a point cloud $P\in {\mathbb{R}}^{N\times 3}$ , an encoder Φ extracts per-point features f=Φ(P) .

To enforce causality, we formalize the following assumptions: let e∈ℰ denote a discrete or continuous environment variable representing domain factors that may influence the observation distribution but are independent of the optimal action a given the task-relevant content. Our goal is to learn a representation f that minimally encodes environment-specific information e that is not already explained by the action a*a*. Formally, we aim to minimize the conditional mutual information I(f;e∣a) .

Since direct computation of I(f;e∣a) is intractable, we derive a variational upper bound via an adversarial environment classifier. Specifically, we introduce a classifier $D_{\psi }(e|f,a)$ that predicts the environment variable e from the representation f and action a . The following variational upper bound holds: $I(f;e|a)\leq E_{p(f,e,a)}[-\log D_{\psi }(e|f,a)]+\text{const}.$

Minimizing this upper bound with respect to the encoder Φ encourages the representation f to be invariant to environment variations. Conversely, maximizing the same quantity with respect to the classifier parameters ψ improves the classifier’s ability to recover e from (f,a) , thus providing a stronger adversarial signal. This forms a standard minimax adversarial game.

To make the optimization directions explicit, we separate the objective into two losses:

Classifier loss (maximizes prediction accuracy, i.e., minimizes cross-entropy):


$$L_{\mathrm{cls}}(\psi )=E_{p\;(f,e,a)}[-\log D_{\psi }(e|f,a)].$$

- Encoder adversarial loss (minimizes predictability of e from f and a ): $L_{\mathrm{enc}}(\Phi )=E_{p\;(f,e,a)}[-\log D_{\psi }(e|f,a)]$ .

In practice, we optimize these objectives in an adversarial minimax fashion (Equation 4):


$$\begin{array}{c} L_{\text{causal}}=\min_{\Phi }\max_{\psi }E_{p\;(f,e,a)}[-\log D_{\psi }(e|f,a)] \end{array}$$ (4)

Where ψ is updated to minimize ${ℒ}_{\mathrm{cls}}$ (i.e., to better predict e from (f,a) ), Φ is updated to maximize the same loss (i.e., to fool the classifier), typically achieved via gradient reversal with a negative sign.

This adversarial procedure explicitly minimizes the mutual information I(e∣f,a) , yielding invariant causal features. To retain task-relevant geometry, we add a reconstruction loss ${ℒ}_{\text{recon}}=∥P-\hat{P}{∥}_{2}^{2}$ where $\hat{P}=\Phi^{-1}(f)$ is a decoded point cloud. The final visual representation is $v=f⊕{\mathrm{MLP}}_{\text{pose}}(q)$ , with q the robot proprioceptive state.

### 3.5 Noise-robust diffusion modeling with confidence-aware denoising

To enhance robustness against observation noise and action stochasticity, we equip the diffusion model with a learnable observation uncertainty estimator. The point cloud encoder outputs both a feature vector f and an uncertainty scalar $\sigma_{\mathrm{obs}}(o)\in {\mathbb{R}}^{+}$ . To ensure numerical stability and interpretable weighting, we constrain $\sigma_{\mathrm{obs}}(o)$ to the range [1,τ] with τ>1(e.g.,τ=2) via a clipped Sigmoid transformation followed by an affine mapping (Equation 5):


$$\begin{array}{c} \sigma_{\mathrm{obs}}(o)=1+(\tau -1)\cdot \text{Sigmoid}(g_{\phi }(o)) \end{array}$$ (5)

Where $g_{\phi }(o)$ is an unbounded scalar output of a small network head. This guarantees $\sigma_{\mathrm{obs}}(o)\in [1,\tau ]$ such that its inverse lies in [1/τ,1] , providing stable mixture weights.

The uncertainty is learned via a heteroscedastic regression formulation. The key idea is to model the prediction error of the encoder’s output as a Gaussian distribution with observation-dependent variance. Let Φ(o) be the encoder output and Φ(o) be a target feature from an exponential moving average (EMA) of the encoder. The EMA provides a temporally smoothed target that reduces stochastic fluctuations, allowing the network to estimate the inherent prediction uncertainty. We minimize the negative log-likelihood of observing the EMA target under a Gaussian distribution with variance $\sigma_{\mathrm{obs}}^{2}(o)$ (Equation 6):


$$\begin{array}{c} L_{\text{uncertainty}}=E_{o\sim D}[\frac{1}{2\sigma_{\mathrm{obs}}^{2}(o)}{\left\|\Phi (o)-\bar{\Phi }(o)\right\|}_{2}^{2}+\frac{1}{2}\log\sigma_{\mathrm{obs}}^{2}(o)] \end{array}$$ (6)

where $\bar{\Phi }(o)$ is an exponential moving average of the encoder.

The first term encourages the encoder to produce features consistent with the EMA target, with the weight inversely scaled by the estimated uncertainty. The second term $\log\sigma_{\mathrm{obs}}^{2}(o)$ prevents the trivial solution where $\sigma_{\mathrm{obs}}(o)\to \infty$ ; it penalizes over-estimation of uncertainty, forcing the model to maintain confidence when predictions are reliable.

We then introduce a confidence-aware denoising network that modulates predictions based on estimated uncertainty. The denoising network is defined as a weighted mixture of a base network and a compensatory network (Equation 7):


$$\begin{array}{c} \begin{array}{l} \in_{\theta }(a^{k},k,v,q)=w\cdot \in_{\theta }^{\text{base}}(a^{k},k,v,q)+\\ \left(1-w)\cdot \in_{\theta }^{\text{comp}}(a^{k},k,v,q\right) \end{array} \end{array}$$ (7)

with the mixture weight defined as $w=\sigma_{\mathrm{obs}}^{-1}(o)\in [1/\tau ,1]$ . Here, $\in_{\theta }^{\text{base}}$ is the primary denoiser used under low uncertainty, while $\in_{\theta }^{\text{comp}}$ handles high-uncertainty scenarios (loww) . The overall noise-robust objective becomes (Equation 8):


$$\begin{array}{c} L_{\text{noise}-\text{robust}}=E_{k,\in ,o}[{\left\|\in -\in_{\theta }(a^{k},k,v,q)\right\|}_{2}^{2}\cdot \sigma_{\mathrm{obs}}^{-2}(o)] \end{array}$$ (8)

The $\log\sigma_{\mathrm{obs}}^{2}$ term from ${ℒ}_{\text{uncertainty}}$ prevents trivial solutions where $\sigma_{\mathrm{obs}}\to \infty$ ,while the multiplication by $\sigma_{\mathrm{obs}}^{-2}(o)$ down-weights the denoising loss for uncertain observations, allowing the model to focus on reliable signals.

### 3.6 Attention anti-interference regularization

Real-world cluttered scenes can distract attention mechanisms. We propose attention anti-interference regularization that penalizes attention on non-task-relevant regions enforces temporal consistency, and is robust to mask inaccuracies.

**Task-relevant masks**: During training, we obtain binary masks $M_{\text{task}}(i)\in \{0,1\}$ for each point $p_{i}$ indicating whether it belongs to a task-relevant object (e.g., cup, bottle, dustbin). These masks are automatically generated from the simulator’s ground-truth semantic labels, which are readily available in our synthetic data generation pipeline without manual annotation. We acknowledge that this constitutes privileged information not directly available in real-world deployments. However, the mask is used only during training to regularize attention behavior; no mask is required at inference time.

For completeness, we also discuss practical alternatives: (1) off-the-shelf foundation models (e.g., SAM) for zero-shot mask prediction, or (2) heuristic masks based on motion cues or bounding boxes from demonstration trajectories. In this work, we rely on simulator-provided masks to isolate the effect of our regularization, but the framework is agnostic to the mask source. Furthermore, to ensure our method is not brittle to imperfect masks, we explicitly design for robustness as described below.

**Mask noise robustness**: To ensure our method is not brittle to imperfect masks, we apply stochastic mask perturbation during training: with probability $p_{\text{perturb}}=0.2$ , we randomly flip the label of a point (from task-relevant to non-task-relevant or vice versa). Additionally, with the same probability, we add Gaussian noise N(0,0.05) to mask boundaries to simulate annotation imprecision. This simulates real-world annotation errors and forces the attention mechanism to rely on geometric structure rather than exact mask boundaries.

The spatial attention regularization penalizes attention on non-task regions (Equation 9):


$$\begin{array}{c} L_{\text{spatial}}=\sum_{i=1}^{N}\alpha_{i}\cdot (1-M_{\text{task}}(i)) \end{array}$$ (9)

Temporal attention consistency enforces stable focus across consecutive steps (Equation 10):


$$\begin{array}{c} L_{\text{temporal}}=\sum_{i=1}^{N}\;|\alpha_{i}^{\left(t\right)}-\alpha_{i}^{\left(t+1\right)}|\cdot M_{\text{task}}(i) \end{array}$$ (10)

We also incorporate stochastic attention masking during training to simulate occlusions: with probability $p_{\text{mask}}=0.15$ , we randomly zero out 20% of the attention weights (sampled uniformly) and renormalize the remaining weights to sum to one. The complete regularization loss is (Equation 11):


$$\begin{array}{c} {ℒ}_{\text{attn}}=\lambda_{\text{spatial}}{ℒ}_{\text{spatial}}+\lambda_{\text{temporal}}{ℒ}_{\text{temporal}} \end{array}$$ (11)

During inference, no masks are required—the learned attention behavior generalizes from the training procedure.

The overall training objective combines the hierarchical diffusion loss, causal representation loss, noise-robust objective, and attention regularization (Equation 12):


$$\begin{array}{c} ℒ={ℒ}_{\text{hier}}+\lambda_{\text{causal}}{ℒ}_{\text{causal}}+\lambda_{\text{noise}}{ℒ}_{\text{noise}-\text{robust}}+\lambda_{\text{attn}}{ℒ}_{\text{attn}} \end{array}$$ (12)

Where $\lambda_{\text{causal}},\lambda_{\text{noise}},\lambda_{\text{attn}}$ are weighting coefficients that balance the contributions of each regularization term. The model is trained end-to-end using the AdamW optimizer with a cosine annealing learning rate schedule.

## 4 Experiments

### 4.1 Experimental setup

**Figure 2:**
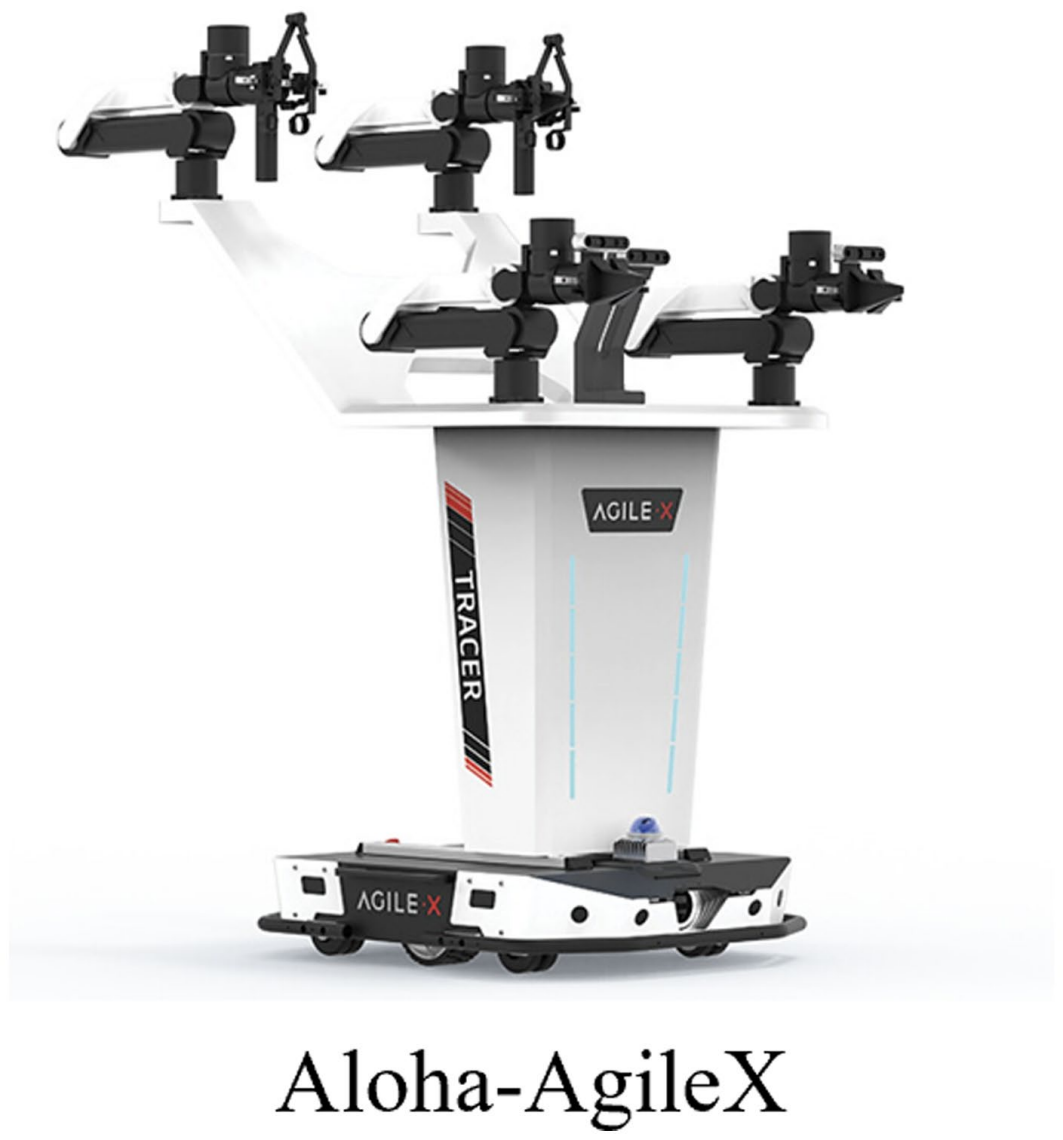
Dual-arm robotic platforms.

**Experimental platform**: All experiments are conducted on the RoboTwin 2.0 platform, which provides a unified simulation environment and a scalable benchmark for bimanual robotic manipulation. Dual-arm evaluations are conducted using Aloha-AgileX platforms, as illustrated in the figure below (Figure 2).

**Data generation**: Training data are automatically synthesized through RoboTwin 2.0’s program-driven task execution pipeline. This generation process leverages multimodal large language models (MLLMs) to produce executable task programs, followed by simulation-in-the-loop refinement with vision-language model (VLM) feedback to ensure trajectory feasibility and task consistency.

**Policy training**: We generate 50 expert demonstrations for each dual-arm configuration. The data format includes multi-view RGB observations, joint states, and action sequences.

**Random seeds**: To ensure statistical reliability, all experiments are conducted on a laptop equipped with an NVIDIA GeForce RTX 4060 GPU (ROG Strix G8 Plus). We use three independent random seeds (42, 100, 1,024) to initialize three separate training runs. These seeds jointly control: (1) network parameter initialization, (2) minibatch shuffling order during training, and (3) domain randomization parameters for each training episode. The same set of seeds is used across all baseline methods and ablation variants to ensure fair comparison. All reported results (mean ± standard deviation) are averaged over the three independent runs seeded accordingly.

**Statistical reporting unit**: All reported results (mean ± standard deviation) are first computed over 100 evaluation rollouts for each of the three independent training runs (seeds). These three run-level statistics are then averaged to obtain the final mean and standard deviation reported in tables and figures. For hypothesis testing, paired *t*-tests are performed at the run/seed level (*N* = 3 paired observations per method-task-setting combination), where the paired unit is the per-seed mean success rate. This avoids the violation of independence assumption that would arise from treating individual rollouts from the same trained policy as independent samples. Bootstrap confidence intervals (1,000 iterations) are computed using seed-level bootstrapping, i.e., resampling the three run-level mean success rates with replacement, rather than pooling rollout-level outcomes. This procedure provides more conservative and statistically appropriate uncertainty estimates for comparisons across methods (Table 1 and 2).

**Table 1:** Hyperparameter configuration for SCH-DIL.

Model and training settings	Hyperparameter	Value		Hyperparameter	Value
Model architecture	Observation steps	3	Training	Learning rate	1e−4
	Action steps	6		Batch size	256
	Prediction horizon T	6		Training iterations	60,000
	Temporal latent horizon	4		Optimizer	AdamW
	Temporal latent dim	32		Weight decay	1e−6
	Diffusion step embed dim	64		LR scheduler	Cosine
	Encoder output dim	128		Warmup steps	5,000
	Down dims	[512, 1,024, 2,048]		Gradient clipping	1.0
	Kernel size	5		Gradient accumulate every	1
	Dropout rate	0.1			
Point cloud encoder	Input channels	3	Random seeds	Seed 1	42
	Output channels	128		Seed 2	100
	PointNet type	PointNet++		Seed 3	1,024
Diffusion model	Train timesteps	100	Loss weights	$\lambda_{\text{casual}}$	0.1
	Inference steps	10		$\lambda_{\text{noise}}$	0.05
	Beta schedule	linear		$\lambda_{\text{attn}}$	0.01
	Beta range	[1e−4, 2e−2]		$\lambda_{\text{spatial}}$	1.0
	Noise schedule	Cosine		$\lambda_{\text{temporal}}$	0.5
Domain randomization configuration	random_background	True	Domain randomization configuration	random_table_height	0.03 (±3 cm)
	clean_background_rate	0.02		random_light	true
	cluttered_table	true		crazy_random_light_rate	0.02

**Table 2:** Model complexity comparison.

Task	Model	Number of parameters	Initial memory (GB)	Peak memory (GB)	Memory increment (GB)	Avg. inference time (s)
Dump bin bigbin	DP3	262.4325 M	2.274	2.353	0.079	1.3632
	OURS	265.6796 M	2.301	2.381	0.080	1.5146
Put bottle dustbin	DP3	262.4325 M	2.274	2.358	0.084	1.6549
	OURS	265.6796 M	2.301	2.387	0.086	1.7984

**Figure 3:**
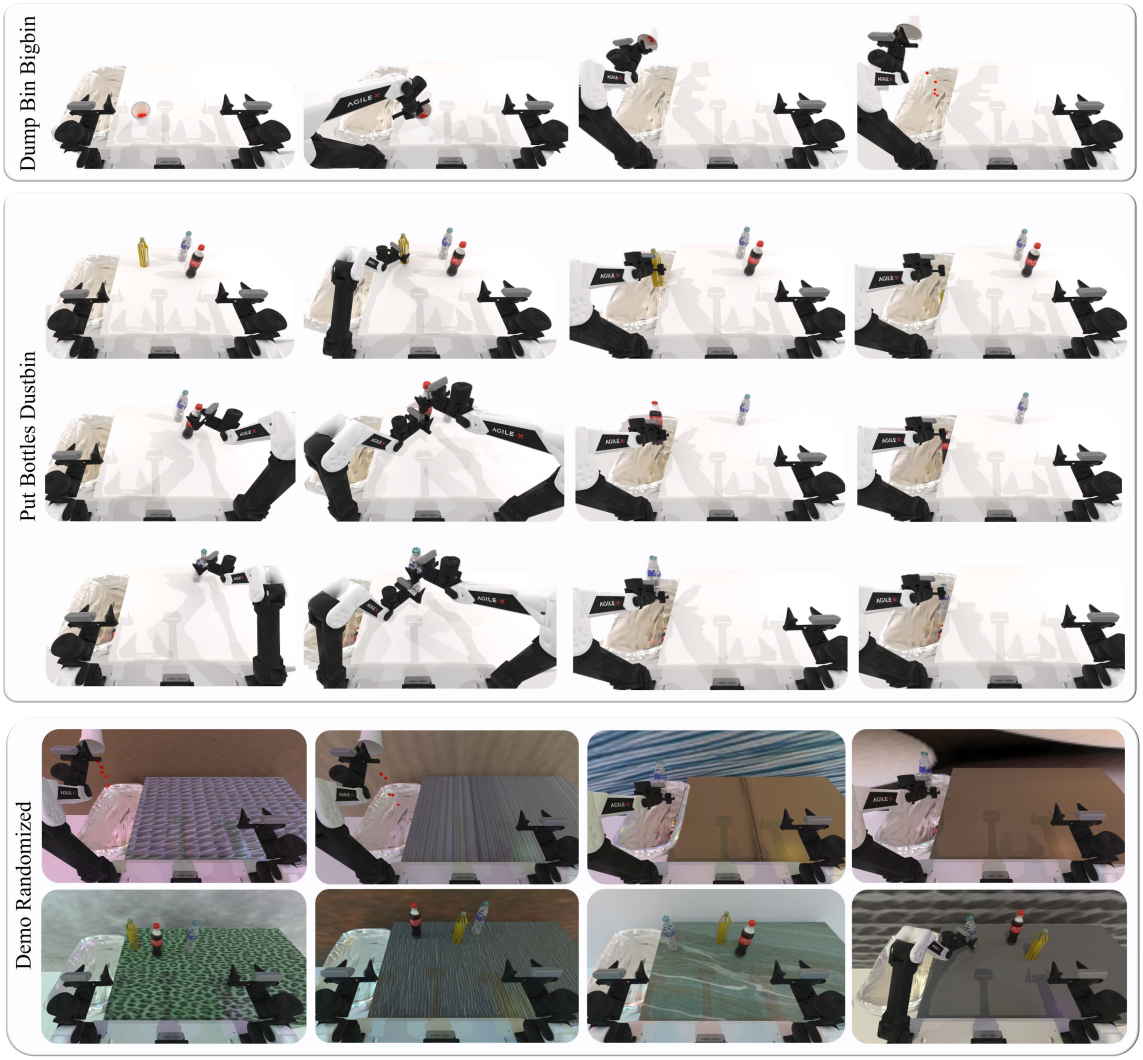
Overview of collaborative bimanual manipulation under clean and domain randomization.

**Task description**: We evaluate our method on representative bimanual manipulation tasks from the RoboTwin 2.0 benchmark, with primary results reported for Dump Bin Bigbin and Put Bottles Dustbin, and extended comparisons on five selected tasks summarized in Figure 3 and Table 3. The full set of tasks evaluated in this study includes: Click Alarmclock, Open Microwave, Place Burger Fries, Open Laptop, and Put Bottles Dustbin. The primary challenges arise from spatial uncertainty of the objects, as well as disturbances in lighting, background conditions, and viewing distance during execution.

**Table 3:** Extended success rate comparison between SCH-DIL and DP3 across all five RoboTwin 2.0 tasks.

Task	DP3 (clean)	SCH-DIL (clean)	DP3 (randomized)	SCH-DIL (randomized)
Click alarm clock	0.75 ± 0.03 [0.69, 0.81]	0.80 ± 0.02 [0.76, 0.84]†	0.13 ± 0.03 [0.07, 0.19]	0.22 ± 0.03 [0.16, 0.28]†
Open microwave	0.60 ± 0.02 [0.56, 0.64]	0.69 ± 0.01 [0.67, 0.71]†	0.21 ± 0.04 [0.13, 0.29]	0.32 ± 0.02 [0.28, 0.36]†
Place burger fries	0.71 ± 0.02 [0.67, 0.75]	0.80 ± 0.02 [0.76, 0.84]†	0.18 ± 0.03 [0.12, 0.24]	0.25 ± 0.02 [0.21, 0.29]†
Open laptop	0.82 ± 0.03 [0.76, 0.88]	0.90 ± 0.02 [0.86, 0.94]†	0.07 ± 0.05 [0.00, 0.17]	0.13 ± 0.04 [0.05, 0.21]†
Put bottles dustbin	0.62 ± 0.02 [0.58, 0.66]	0.71 ± 0.01 [0.69, 0.73]†	0.25 ± 0.03 [0.19, 0.31]	0.39 ± 0.02 [0.35, 0.43]†
Average	0.70 ± 0.03 [0.65, 0.76]	0.78 ± 0.02 [0.75, 0.82]	0.17 ± 0.04 [0.10, 0.22]	0.26 ± 0.03 [0.21, 0.30]

Means and standard deviations are computed by first averaging over 100 rollouts per seed (3 seeds), then reporting the mean and standard deviation across seeds. Paired *t*-tests (†) are conducted at the seed level (*N* = 3) comparing per-seed mean success rates between SCH-DIL and DP3. Bootstrap confidence intervals (1,000 iterations) are computed using seed-level resampling.

Results are reported as mean ± standard deviation over 100 rollouts per seed, averaged across three independent training runs. Values in brackets are 95% confidence intervals (bootstrap, 1,000 iterations). † indicates *p* < 0.01 compared to DP3 (paired *t*-test).

### 4.2 Implementation details

To ensure reproducibility, this section details the experimental configuration, including the evaluation protocol and hyperparameter settings used in our study.

**Evaluation protocol**: All methods are evaluated under identical conditions within the same simulation environment to guarantee fairness and consistency. For each task, we conduct 100 independent rollouts per random seed and report the mean success rate ± standard deviation, averaged across three independent training runs with different seeds. All experiments are performed on a laptop equipped with an NVIDIA GeForce RTX 4060 GPU (ROG Strix G8 Plus).

**Model hyperparameters**: We provide a comprehensive description of the hyperparameter configurations for the core model used in our experiments. The following table summarizes the key hyperparameters optimized for our dual-arm manipulation tasks.

**Additional hyperparameters for robust components**: The attention anti-interference regularization (Section 3. D) uses the following hyperparameters: mask flip probability $p_{\text{perturb}}=0.2$ , attention masking probability $p_{\text{perturb}}=0.15$ , attention drop fraction 0.2 (i.e., 20% of attention weights are zeroed out when masking is applied), and mask boundary noise standard deviation 0.05 for simulating annotation imprecision. The noise-robust diffusion modeling (Section 3. C) uses τ=0.2 to bound the observation uncertainty $\sigma_{\mathrm{obs}}(o)\in [1,\tau ]$ .

### 4.3 Experimental results

**Figure 4:**
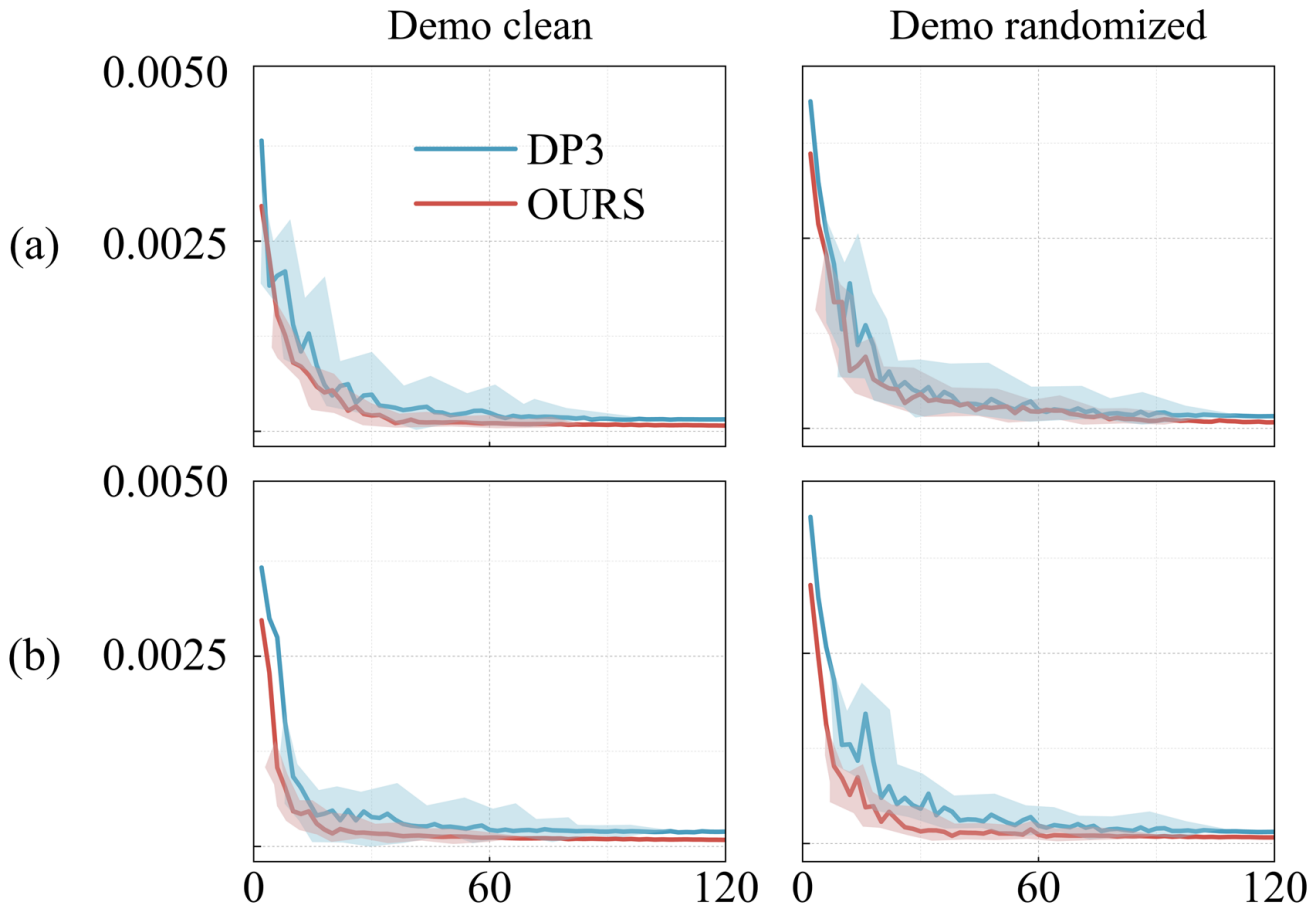
Training convergence curves for **(a)** Dump Bin Bigbin and **(b)** Put Bottles Dustbin tasks.

**Validation loss analysis**: We monitored the validation loss throughout the training process to evaluate the convergence behavior and training stability of our proposed method. Figure 4 presents the convergence trend of the validation loss curves recorded across independent training runs.

The proposed method exhibits smooth and stable convergence for our method, with minimal oscillations in the later stages of training.

**Computational complexity analysis**: We evaluate the computational efficiency of our method by comparing its memory usage and inference latency with baseline.

Our approach introduces minimal memory overhead while delivering stronger task performance, maintaining a practical balance between robustness and real-time feasibility for bimanual manipulation.

**Figure 5:**
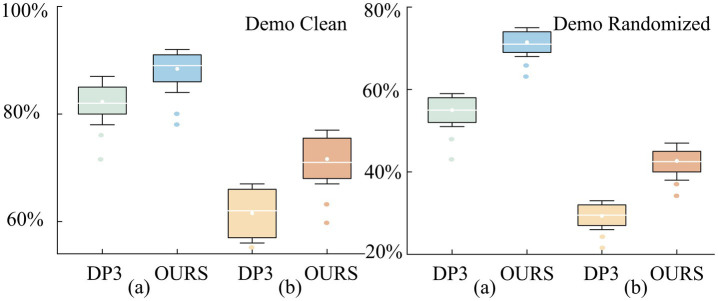
Comparative analysis of success rates on **(a)** Dump Bin Bigbin and **(b)** Put Bottles Dustbin tasks under distinct inference environment settings.

**Task success rate evaluation**: Figure 5 presents the validation results of task success rates for models trained using the aforementioned method. Each task was evaluated using identical datasets and inference environments across two distinct settings: Demo Clean and Demo Randomized. All improvements of our method over DP3 reported in Tables 3 and 4 are statistically significant (*p* < 0.01, paired *t*-test, run-level, *N* = 3). While some 95% confidence intervals in Table 3 exhibit partial overlap (e.g., Open Laptop under randomized settings), the paired *t*-test accounts for the correlation between method performances on the same seeds and confirms significance.

**Table 4:** Success rate comparison across different methods and tasks.

Method	Dump bin bigbin (demo clean)	Dump bin bigbin (demo randomized)	Put bottles dustbin (demo clean)	Put bottles dustbin (demo randomized)
RDT	0.64 ± 0.03 [0.58, 0.70]	0.32 ± 0.04 [0.24, 0.40]	0.21 ± 0.03 [0.15, 0.27]	0.04 ± 0.02 [0.00, 0.08]
Pi0	0.83 ± 0.02 [0.79, 0.87]	0.24 ± 0.03 [0.18, 0.30]	0.54 ± 0.03 [0.48, 0.60]	0.13 ± 0.03 [0.07, 0.19]
ACT	0.68 ± 0.03 [0.62, 0.74]	0.01 ± 0.01 [0.00, 0.03]	0.27 ± 0.03 [0.21, 0.33]	0.01 ± 0.01 [0.00, 0.03]
DP	0.49 ± 0.03 [0.43, 0.55]	0.00 ± 0.00 [0.00, 0.00]	0.22 ± 0.03 [0.16, 0.28]	0.00 ± 0.00 [0.00, 0.00]
DP3	0.81 ± 0.02 [0.77, 0.85]	0.53 ± 0.03 [0.47, 0.59]	0.62 ± 0.02 [0.58, 0.66]	0.25 ± 0.03 [0.20, 0.32]
OURS	0.90 ± 0.01 [0.88, 0.92]†	0.68 ± 0.02 [0.64, 0.72]†	0.71 ± 0.01 [0.69, 0.73]†	0.39 ± 0.02 [0.36, 0.44]†

Means and standard deviations are computed by first averaging over 100 rollouts per seed (3 seeds), then reporting the mean and standard deviation across seeds. Paired *t*-tests (†) are conducted at the seed level (*N* = 3) comparing per-seed mean success rates between SCH-DIL and DP3. Bootstrap confidence intervals (1,000 iterations) are computed using seed-level resampling.

Results are reported as mean ± standard deviation over 100 rollouts per seed, averaged across three independent training runs. Values in brackets are 95% confidence intervals computed via bootstrap resampling (1,000 iterations). † indicates *p* < 0.01 compared to DP3 (paired *t*-test).

Across both tasks and inference environment settings, our method consistently outperforms the baseline, achieving higher median success rates and demonstrating enhanced stability and effectiveness in bimanual manipulation scenarios.

**Figure 6:**
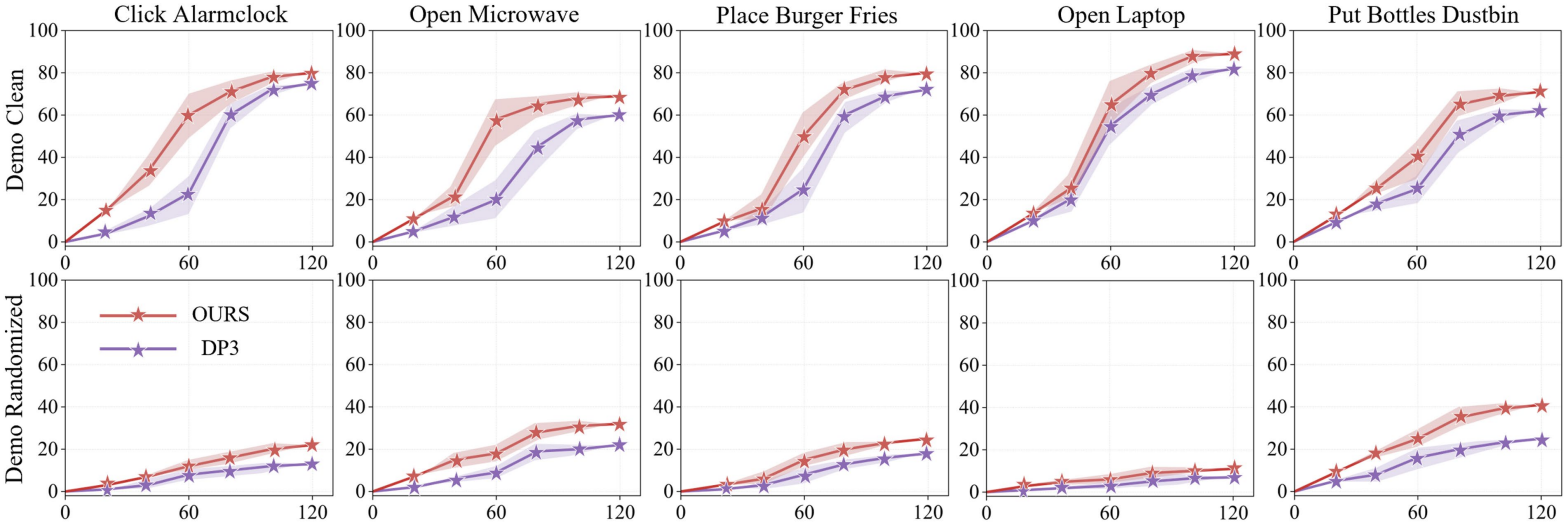
Comparative success rates of SCH-DIL and DP3 across five bimanual tasks under clean and domain-randomized inference settings. Results are reported as mean success rates over 100 rollouts per seed, averaged across three independent training runs.

**Extended comparison across five tasks**: To further assess generalization across a broader set of bimanual behaviors, we compare SCH-DIL against the strongest baseline DP3 on five selected tasks from RoboTwin 2.0. Figure 6 and Table 3 reports this extended comparison. All results in Figure 6 are reported as mean success rates with standard deviation error bars, computed over 100 evaluation rollouts per seed and aggregated across three independent training runs. As shown in Figure 6 and Table 3, SCH-DIL consistently achieves higher mean success rates than DP3 on all five selected tasks under both clean and domain-randomized evaluation conditions. The expanded five-task comparison is conducted only against DP3, as it represents the strongest baseline in most settings and enables a focused evaluation of our method’s generalization across tasks.

As summarized in Table 4, we further assess the efficacy of our proposed method by benchmarking it against several baseline approaches. All methods are evaluated under identical conditions. To ensure statistical reliability, results are reported as mean ± standard deviation over 100 rollouts per seed, averaged across three independent training runs with different random seeds.

**Ablation studies**: To systematically investigate the contribution of each core component in SCH-DIL, we conduct comprehensive ablation experiments on two representative tasks: Dump Bin Bigbin and Put Bottles Dustbin. All ablations are evaluated under the same protocol as the main experiments, reporting mean success rates over 100 rollouts per seed averaged across three independent training runs. Table 5 presents the ablation results under both clean and domain-randomized inference settings.

**Table 5:** Ablation study results: success rates across different component configurations.

Variant	Dump bin bigbin (clean)	Dump bin bigbin (randomized)	Put bottles dustbin (clean)	Put bottles dustbin (randomized)
w/o Hierarchical	0.86 ± 0.02 [0.82, 0.90]	0.63 ± 0.02 [0.59, 0.67]	0.67 ± 0.02 [0.63, 0.71]	0.34 ± 0.03 [0.28, 0.40]
w/o Causal	0.87 ± 0.01 [0.85, 0.89]	0.60 ± 0.03 [0.54, 0.66]	0.69 ± 0.02 [0.65, 0.73]	0.31 ± 0.02 [0.27, 0.35]
w/o Noise-robust	0.87 ± 0.02 [0.83, 0.91]	0.66 ± 0.02 [0.62, 0.70]	0.68 ± 0.02 [0.64, 0.72]	0.37 ± 0.03 [0.31, 0.43]
w/o Attention	0.87 ± 0.01 [0.85, 0.89]	0.63 ± 0.02 [0.59, 0.67]	0.69 ± 0.02 [0.65, 0.73]	0.34 ± 0.03 [0.28, 0.40]
w/o Temporal branch	0.84 ± 0.02 [0.80, 0.88]	0.57 ± 0.03 [0.51, 0.63]	0.65 ± 0.03 [0.59, 0.71]	0.27 ± 0.04 [0.19, 0.35]
w/o Spatial branch	0.85 ± 0.02 [0.81, 0.89]	0.58 ± 0.02 [0.54, 0.62]	0.65 ± 0.02 [0.61, 0.69]	0.28 ± 0.03 [0.22, 0.34]
w/ Noisy mask	0.88 ± 0.02 [0.84, 0.92]	0.65 ± 0.03 [0.59, 0.71]	0.69 ± 0.02 [0.65, 0.73]	0.37 ± 0.03 [0.31, 0.43]
Full SCH-DIL	0.90 ± 0.01 [0.88, 0.92]	0.68 ± 0.02 [0.64, 0.72]	0.71 ± 0.01 [0.69, 0.73]	0.39 ± 0.02 [0.36, 0.44]

Results are reported as mean ± standard deviation over 100 rollouts per seed, averaged across three independent training runs. Values in brackets are 95% confidence intervals (bootstrap, 1,000 iterations).

Several observations emerge from the ablation results. Removing either the temporal or spatial branch leads to substantial performance degradation. The causal module contributes noticeably under domain shifts, with performance dropping sharply when removed under randomized conditions. All ablation variants consistently outperform the DP3 baseline, confirming that each proposed component provides a positive contribution, and their integration in the full SCH-DIL model achieves the best overall results.

## 5 Conclusion

In this paper, we propose SCH-DIL (Spatiotemporal Causal Hierarchical Diffusion Imitation Learner), a novel visuomotor imitation learning framework designed for long-horizon generalizable bimanual manipulation. The method introduces four key technical contributions: spatiotemporal hierarchsical diffusion optimization that factorizes action generation into temporal and spatial denoising processes for multi-scale modeling; causal visual representation learning that minimizes mutual information with confounding environmental factors to produce invariant features; noise-robust diffusion modeling with observation uncertainty estimation and confidence-aware denoising; and attention anti-interference regularization that penalizes distractions and enforces temporal attention consistency. Extensive experiments on the RoboTwin 2.0 benchmark demonstrate that SCH-DIL consistently outperforms existing diffusion-based and imitation learning baselines, achieving higher success rates under both clean and domain-randomized inference settings with minimal computational overhead. The results validate that integrating hierarchical spatiotemporal modeling, causal representation learning, and robust attention mechanisms into a unified diffusion policy framework significantly enhances generalization, robustness, and higher success rates for bimanual visuomotor imitation.

### 5.1 Limitations and future work

Our method currently relies on ground-truth semantic masks and known environmental confounders during training, which increases training complexity, and has only been evaluated in simulation. To bridge the simulation-to-real gap, future work will explore several concrete directions. Self-supervised mask prediction methods, such as using foundation models like SAM to generate pseudo-masks without manual annotation, or training a lightweight mask predictor from motion cues and bounding boxes derived from demonstration trajectories, will be investigated. Domain adaptation techniques will be explored to align simulated and real visual features, reducing the reliance on perfect mask annotations during training. The approach will also be extended to real-robot platforms, starting with controlled lab environments and gradually increasing scene complexity. Additionally, future work will focus on further assessing computational efficiency through a systematic comparison with diffusion acceleration methods, reducing training overhead, and extending the approach to cross-embodiment settings.

## Data Availability

The raw data supporting the conclusions of this article will be made available by the authors, without undue reservation.
